# Enhanced and Stem-Cell-Compatible Effects of Nature-Inspired Antimicrobial Nanotopography and Antimicrobial Peptides to Combat Implant-Associated Infection

**DOI:** 10.1021/acsanm.2c04913

**Published:** 2023-02-15

**Authors:** Mohd Irill Ishak, Marcus Eales, Laila Damiati, Xiayi Liu, Joshua Jenkins, Matthew J. Dalby, Angela H. Nobbs, Maxim G. Ryadnov, Bo Su

**Affiliations:** † Bristol Dental School, University of Bristol, Bristol BS1 2LY, U.K.; ‡ National Physical Laboratory, Teddington TW11 0LW, U.K.; § Centre for the Cellular Microenvironment, University of Glasgow, Glasgow G11 6EW, Scotland; ∥ Department of Biology, College of Science, University of Jeddah, Jeddah 23218, Saudi Arabia

**Keywords:** titanium, implants, bacteria, nanotopography, antimicrobial peptide

## Abstract

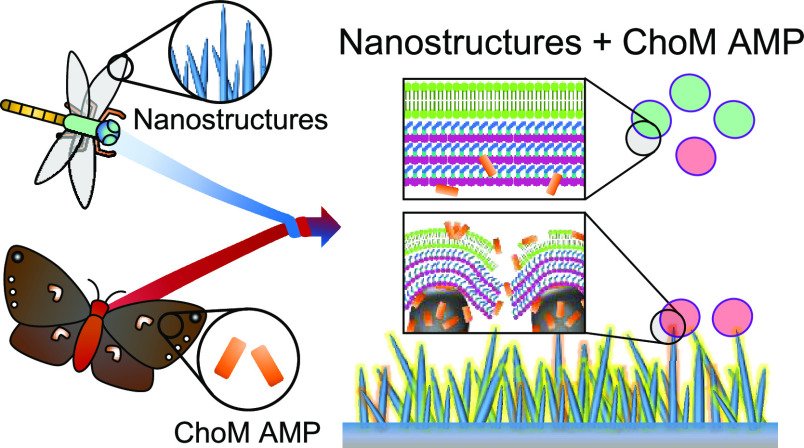

Nature-inspired antimicrobial surfaces and antimicrobial peptides (AMPs) have emerged as promising strategies to combat implant-associated infections. In this study, a bioinspired antimicrobial peptide was functionalized onto a nanospike (NS) surface by physical adsorption with the aim that its gradual release into the local environment would enhance inhibition of bacterial growth. Peptide adsorbed on a control flat surface exhibited different release kinetics compared to the nanotopography, but both surfaces showed excellent antibacterial properties. Functionalization with peptide at micromolar concentrations inhibited *Escherichia coli* growth on the flat surface, *Staphylococcus aureus* growth on the NS surface, and *Staphylococcus epidermidis* growth on both the flat and NS surfaces. Based on these data, we propose an enhanced antibacterial mechanism whereby AMPs can render bacterial cell membranes more susceptible to nanospikes, and the membrane deformation induced by nanospikes can increase the surface area for AMPs membrane insertion. Combined, these effects enhance bactericidal activity. Since functionalized nanostructures are highly biocompatible with stem cells, they make promising candidates for next generation antibacterial implant surfaces.

## 1 Introduction

Titanium dental and orthopedic implants are an essential component of modern medical treatments. Dental implants replace missing teeth resulting from trauma or periodontal disease, while orthopedic implants replace joints such as the hip and knee as a treatment for chronic diseases such as osteoarthritis.^1−4^ The use of these implants has increased rapidly due to aging populations and rising obesity levels.^3^ Bacterial infection is one of the most common causes of premature implant failure, and the most prevalent microbes associated with orthopedic implant infections are Gram-positive staphylococci, particularly *Staphylococcus aureus* and *Staphylococcus epidermidis*, which account for 80% of all implant infections, and the Gram-negative bacteria *Pseudomonas aeruginosa*, *Klebsiella pneumoniae*, and *Escherichia coli*.^5^ The subsequent revision surgery required upon implant failure has serious potential ramifications for the patient and places a significant burden on the healthcare infrastructure, and with increasing bacterial antimicrobial resistance, the infections are becoming more difficult to treat.^6,7^ Alternative strategies to combat implant-associated infections are therefore of great clinical need.

Nanotopographies exhibiting antifouling or bactericidal properties have been observed across the natural world from lotus leaves to shark skin and butterfly wings.^8^ Previous studies have succeeded in growing analogous nanotopographies on titanium, and some have reported bactericidal effects, especially against motile and Gram-negative species, such as *P. aeruginosa* and *E. coli*.^9,10^ This presents the opportunity to generate novel antimicrobial implant materials by exploiting nature-inspired nanotopographies. Similarly, as antimicrobial resistance levels are escalating against the last-resort antibiotics and newest-generation drugs, alternatives are being pursued such as antimicrobial peptides (AMPs). AMPs are part of the innate immune system of bacteria, archaea, protists, fungi, plants, and animals. AMPs have been shown to exhibit bactericidal activity against multidrug resistant bacteria, highlighting their potential as promising alternatives to current antimicrobials that have become redundant due to the rise in resistance.^11,12^ When the amino acid sequence of an AMP is known, it may be synthesized using approaches such as solid phase peptide synthesis (SPPS), and its activity may be improved by amino acid modification.^13^

The AMP used in this study is a specifically designed adaptation of the naturally occurring AMP cecropin B (CecB),^14^ first isolated from the haemolymph of the giant silk moth, *Hyalophora cecropia*. CecB has known activity against Gram-negative bacteria, such as *P. aeruginosa* and *E. coli*, but little or no activity reported against Gram-positive bacteria, such as *S. aureus*.^15,16^ To increase the spectrum of bactericidal activity for CecB, modifications were designed by Pfeil et al. resulting in a chopped cecropin mutant (ChoM), which proved to be effective against both Gram-positive and Gram-negative bacteria.^16^

In this work, we fabricated nature-inspired nanospike (NS) surfaces using pure titanium and explored the capacity for their antibacterial effects to be enhanced by the incorporation of ChoM, with the goal of generating an antibacterial and biocompatible biomaterial that is effective against both Gram-negative and Gram-positive bacteria.

## 2 Experimental Procedures

### 2.1 Formation of Titanium Oxide Nanospikes on Pure Titanium Substrate via Alkaline Hydrothermal Method

Titanium (Grade 1) disks were polished to a mirror shine to obtain <10 nm roughness across the disk to optimize NS orientation during the subsequent alkaline hydrothermal growth. The disks were polished on a Struers TegraPol-15 with silicon carbide grinding paper (Struers) at increasing grit levels from 80 to 4000 on MD Fuga pads (Struers) at 30 N and 300 RPM for 4 min each. To obtain a mirror shine, the disks were polished with MD Chem pads (Struers) at 35 N and 150 RPM and 10% hydrogen peroxide (Acros Organics) in colloidal silica suspension (Struers) for 15 min. The disks were cleaned by sonication (Grant XUB5) for 15 min in deionized water, preheated to 40 °C, and immersed in absolute ethanol (Merck) for 10 min before blow-drying with compressed air.

Polished titanium disks (24) were slotted into custom-made PTFE holders to ensure the disks remained upright and placed into a 125 mL PTFE cup. The cup was then inserted into an acid digestion vessel (Parr Instrument Company-Model 4748) containing 52 mL of 1 M NaOH (Fisher). The vessel was tightly sealed and placed in a preheated oven (Gallenkamp Plus II) for 2 h at 240 °C. After the alkaline hydrothermal treatment, the acid digestion vessel was removed from the oven and left to cool to room temperature. The disks were then removed from the holders and soaked in deionized water and absolute ethanol for 10 min each. The disks were finally placed on ceramic blocks and left to dry overnight.

**Figure 1:**
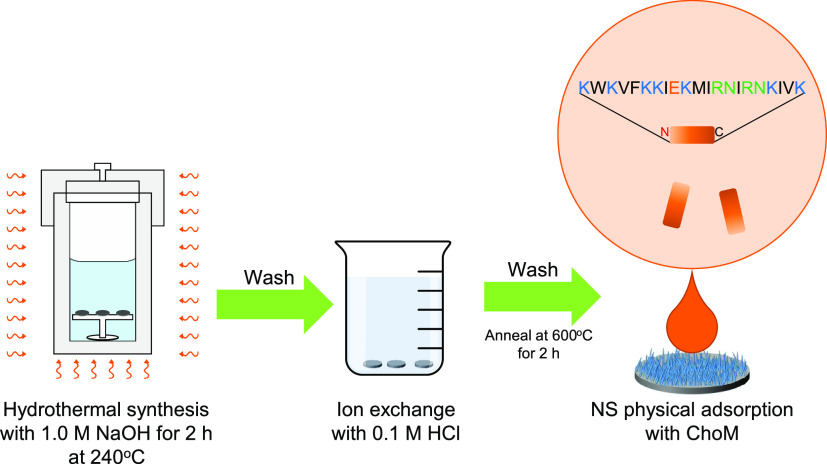
Schematic illustration of the fabrication of TiO_2_ nanospikes (NS) and functionalization with ChoM. The inset shows the ChoM peptide sequence.

After the alkaline hydrothermal treatment, the disks were initially heated at 300 °C (temperature ramp of 10 °C/min) for 1 h using a chamber furnace (Elite Thermal Systems Ltd., Model-BMF 11/7) to ensure the nanospikes fixed to the titanium disk surface. When cooled, the disks were immersed in 0.6 M HCl (Fisher) for 1 h where the sodium in the nanospikes was exchanged with the hydrogen in the HCl to form hydrogen titanate. The disks were then rinsed with deionized water and absolute ethanol for 10 min each and air dried. The final step involved placing the disks in the chamber furnace for calcination for 2 h at 600 °C where the hydrogen titanate nanospikes were converted into TiO_2_. The disks were cooled and stored in a sterile, enclosed plastic Petri dish until use (Figure 1).

### 2.2 Scanning Electron Microscopy (SEM)

Samples were prepared for SEM by sputter coating (Emitech K757X) the surface with a conductive metal layer of ∼6 nm thick consisting of 20% palladium and 80% gold. The samples were imaged on a FEI Quanta 200 scanning electron microscope at various magnifications.

### 2.3 X-ray Photoelectron Spectroscopy (XPS)

The surface elemental composition of mirror polished pure titanium disks and NS disks was analyzed by XPS in ultrahigh vacuum (UHV) setup equipped with a high-resolution specs PHOIBOS 150 2D-DLD elevated pressure energy analyzer equipped with differential pumping system. A monochromatic Al Kα X-Ray source was used with a photon energy of 1486.6 eV and anode operating energy of 15 kV. The base pressure was ∼2 × 10^–10^ bar. A survey scan (settings of 0.5 eV steps, 0.1 s dwell time, epass 40, and range between 1100 and −10 eV) was initially performed to determine the elemental peaks in the sample. Ti2p, O1s, C1s, and N1s peaks were observed. Peaks were fitted using the CasaXPS software.

### 2.4 Bacterial Interactions with Nanospikes

Mueller–Hinton broth cultures (10 mL) were incubated for 16 h at 37 °C and 220 RPM. These were then subcultured into 20 mL of prewarmed broth in a 50 mL conical flask to an optical density at 600 nm (OD_600_) of 0.1 and further incubated at 37 °C and 220 RPM until the start of exponential phase growth (usually after 1.5–3 h). Bacterial suspensions were then adjusted to the desired cell density of 10^6^ CFU/mL in MH broth. Details of the strains can be found in Table S1.

Prior to bacterial inoculation, disks were immersed in absolute ethanol for 10 min within a sterile, plastic Petri dish, thoroughly washed with 0.01 M Tris–HCl, and then air dried within a flow hood (Brassaire). Once dried, the disks were stored in sterile Petri dishes until utilized.

#### 2.4.1 Live/Dead Staining

*Bac*Light live/dead staining (Invitrogen) was used to investigate the membrane integrity of adherent bacteria on the NS surfaces. The surface of each disk was inoculated with 40 μL of bacterial suspension, incubated at 37 °C for 3 h, and then gently washed with Tris–HCl. SYTO9/Propidium iodide was prepared according to manufacturer’s instructions and 40 μL applied to each surface and left for 15 min in the dark at ambient room temperature. Disks were then washed twice with Tris–HCl to remove excess stain. The disks were placed onto a glass slide and covered with a glass cover slip and imaged under a fluorescence microscope at wavelengths 450–490 and 515–560 nm. The relative numbers of bacterial cells with intact membranes (fluorescing green) and membrane-compromised cells (fluorescing red) were quantified using Image J (NIH) software.

#### 2.4.2 BacTiter-Glo

Aliquots (40 μL) of bacterial suspension were applied to the surface of flat and NS disks within a white, opaque 24-well plate (Perkin Elmer) and incubated within a humidity chamber at 37 °C for 0.5–3 h. BacTiter-Glo reagent (40 μL) was added to the bacterial suspension and the luminescence was measured in a plate reader (Tecan Infinite F200 Pro) with automatic attenuation and 1000 ms integration time.

#### 2.4.3 RealTime-Glo

RealTime-Glo assay (Promega) was used since it allows continuous monitoring of the metabolic activity of mammalian or bacterial cells and has particularly good sensitivity for Gram-positive bacteria.^17^ Bacterial suspensions (1 mL) were mixed with 1 μL of MT cell viability substrate and 1 μL of NanoLuc enzyme, and incubated in the dark for 1 h at 37 °C and 220 RPM. Bacterial suspensions (40 μL) were then applied to disks within a white, opaque 24-well plate, which was then sealed with transparent film (Greiner Bio-one EasySeal plate sealer) to ensure sterility and to prevent the surfaces from drying out. The plate was placed in a preheated (37 °C) plate reader (Tecan infinite F200 Pro) and luminescence recorded every 10 min for up to 18 h with 1000 ms integration time, wait time of 0.1 s, and settle time of 150 ms.

### 2.5 ChoM Biofunctionalization

Aliquots of ChoM (KWKVFKKIEKMIRNIRNKIVK-am) at three different concentrations (25, 50, and 100 μM, 40 μL) were applied to disks under sterile conditions (within a flow hood) until visually dried (typically around 3 h). The disks were stored at 4 °C until required (Figure 1).

### 2.6 ChoM Release Quantification Using Nanodrop

The release of ChoM from flat and NS disks was quantified using the Nanodrop (SimpliNano) at 280 nm. Deionized water (40 μL) was applied to the surface of each disk and incubated at 37 °C for a determined time duration. Aliquots (2 μL) were transferred at periodic intervals to the Nanodrop for *A*_280_ measurements. At the determined time interval, the broth was removed from the disk and the disks were left to dry before being processed for SEM.

### 2.7 Cell Culture

Human mesenchymal stem cells (hMSCs) (Promocell) were cultured in Dulbecco’s modified eagle medium (DMEM) (Sigma-Aldrich) supplemented with 1% penicillin/streptomycin (Invitrogen), 1% (v/v) l-glutamine (200 mM, Gibco), 1% sodium pyruvate (Sigma-Aldrich), 1% nonessential amino acids (Sigma-Aldrich), and 2% antibiotics (6.74 U/mL penicillin–streptomycin, 0.2 μg/mL fungizone; Sigma), and 10% foetal bovine serum (FBS) (Invitrogen) at 37 °C in 5% CO_2_. No cells beyond passage 4 were used. Seeding on titanium disks was done in 24-well plates at 10^4^ cells/disk, with 5% FBS. The culture medium was replenished every 3 days for up to 28 days. For Geimsa staining studies, osteogenic medium (MERCK, Germany) was used as a positive control.

### 2.8 AlamarBlue Assay

AlamarBlue solution (Bio-Rad) was mixed 1:10 in DMEM, and 900 μL of it was applied to each titanium disk before incubating for 6 h at 37 °C in 5% CO_2_. Aliquots (200 μL) were transferred in triplicate into a 96-well plate and analyzed with a Thermo Scientific Multiskan FC. Absorbance was measured at *A*1 = 570 and *A*2 = 600 nm.

### 2.9 Immunofluorescence Staining

hMSCs were seeded onto the disks at a cell density of 3000 cells/cm^2^ and incubated for 3 days. Then, the samples were washed with PBS (Sigma-Aldrich), fixed for 15 min at 37 °C with 3.7% v/v formaldehyde/PBS, permeabilized, and stained for vinculin using monoclonal antivinculin antibody (1:100 dilution) (Sigma-Aldrich), and phalloidin-rhodamine actin diluted 1:500 in PBS/BSA. The antibody was removed, and the cells were washed three times for 5 min in PBS/0.5% v/v Tween. A secondary antibody (horse anti-mouse IgG, biotinylated, Vector Labs, U.K., Z0715) diluted 1:50 PBS/BSA was added for 1 h at 37 °C. The antibody was removed, and the cells were washed three times for 5 min in PBS/0.5% v/v Tween. Streptavidin-FITC (Vector Laboratories, U.K., SA-5001) was diluted in 1:50 PBS/BSA and incubated for 30 min at 4 °C. Disks were rinsed three times for 5 min in PBS/0.5% v/v Tween. Visualization was via a fluorescence microscope (Zeiss Axiovert 200 M, 10× magnification, NA 0.5). Comparisons of staining intensity between surfaces were analyzed using Image J software version 1.42q.

### 2.10 Giemsa Staining

For the histological analysis of cells on titanium surfaces, Giemsa staining was used. After cell fixation (as above), the cells were stained with Giemsa stock solution (MERCK, Germany) for 1 min and washed thoroughly with distilled water. The samples were air dried and observed using a Zeiss immunofluorescence microscope at 495/519 nm and under normal light. This staining method allowed visualization of the cell nuclei and cytoplasm, providing information on cell morphology and organization on the titanium surfaces.

### 2.11 Statistical Analyses

All statistical analyses were performed using GraphPad Prism V9. Data were analyzed by ANOVA with the Tukey HSD post hoc test, and *p*-values <0.05 were considered significant. Unless otherwise stated, values given are mean ± standard deviation and are representative of three experimental replicates (*n* = 3) performed in duplicate.

## 3 Results

### 3.1 Functionalization of Titanium Surfaces with ChoM

**Figure 2:**
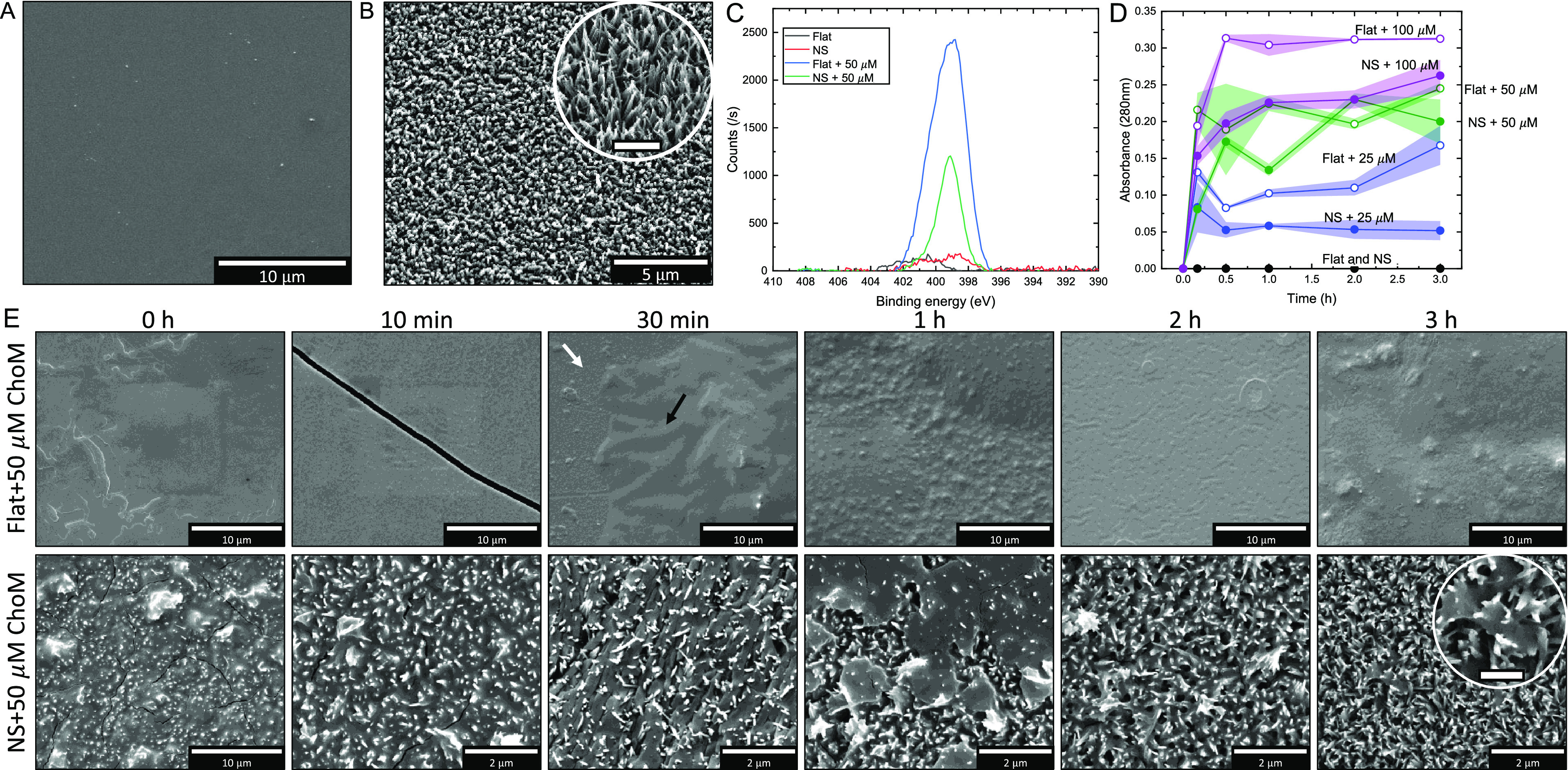
Characterization of ChoM functionalization of titanium surfaces. SEM image of nonfunctionalized (A) flat titanium disk and (B) NS disk (inset scale bar is 1 μm). (C) XPS spectra of the functionalized (50 μM total peptide) and nonfunctionalized flat and NS surfaces. (D) Release profiles from flat and NS surfaces over 3 h as determined by absorbance at 280 nm. (E) SEM images of ChoM (50 μM total peptide) coating on flat titanium (top) and NS (bottom) surfaces following elution into MH broth over a 3-h period. White and black arrows on Flat + 50 μm ChoM after 30 min show the bare disk and the coated area, respectively. Inset scale bar of NS + 50 μm ChoM after 3 h is 500 nm. Data are presented as mean ± SD, *n* = 2.

Flat titanium (control) and NS surfaces were functionalized with ChoM through physical adsorption, whereby 40 μL of ChoM in deionized water was left to dry onto the surfaces. Peptide was adsorbed onto the surfaces at increasing micromolar concentrations above its MIC values to compensate for the potential loss of the peptide from the surface and to establish an optimal concentration for surface functionalization (25 M, 50 M, and 100 μM). SEM was used to assess the homogeneity of initial peptide coatings on the titanium surfaces and over a 3-h elution period (Figure 2A,B,E). The flat surfaces showed more homogenous or larger peptide deposits with only some of the coating remaining visible 30 min into the elution period. By contrast, the peptide coverage on the NS surfaces appeared to lack homogenous distribution with some NS areas covered with the peptide material more visibly than others. Much of the peptide coating on the NS surfaces remained visible after a 10-min elution period. Only the tips of the nanospikes could be visualized where the coating was thick. The peptide functionalized surfaces were further characterized using XPS to confirm peptide presence by detecting the nitrogen peak at 399 eV (Figure 2C). The peak corresponds to the amine group and was detected on both flat and NS functionalized surfaces, confirming the presence of ChoM. It was also found that the relative atomic percentage of amine groups on the flat surfaces was significantly higher than on the NS surfaces, suggesting that more peptide was retained on the flat surfaces compared to on the NS surfaces. Our XPS analysis also confirmed that the nanospikes consisted of pure titanium dioxide and a small amount of metal hydroxides (Supplementary Figure 1). No sodium from the sodium titanate was present following the ion exchange treatment with HCl before annealing, but the presence of −OH groups on the nanospikes was expected.

The ChoM release profiles for each surface across the concentration range were determined by measuring absorbance of the eluate at 280 nm. This wavelength is used to detect aromatic residues in peptides, of which in ChoM, there are two (tryptophan and phenylalanine). For the flat surfaces, there was a rapid release of peptide into the MH broth within the first 30 min and once reached, the maximum absorbance signal was maintained over the 3-h period (Figure 2D). Based on these data and the SEM observations, it was estimated that >90% of the AMPs were released from the flat surface after 3 h. In comparison, the NS surface generally showed a lower overall level of peptide release but increases still occurred beyond 1 h in a dose-dependent manner, indicating a more gradual peptide release after the initial 10-min period. The exception to this was for the NS disk functionalized with 25 μM ChoM where no gradual increase in absorbance was observed after the initial 10-min release.

### 3.2 Bactericidal Activity of Peptide-Functionalized Surfaces

Having demonstrated the release of ChoM into the local environment, the next step was to determine the antibacterial properties of the functionalized NS surface. The control used in this study was the nonfunctionalized flat surface.

**Figure 3:**
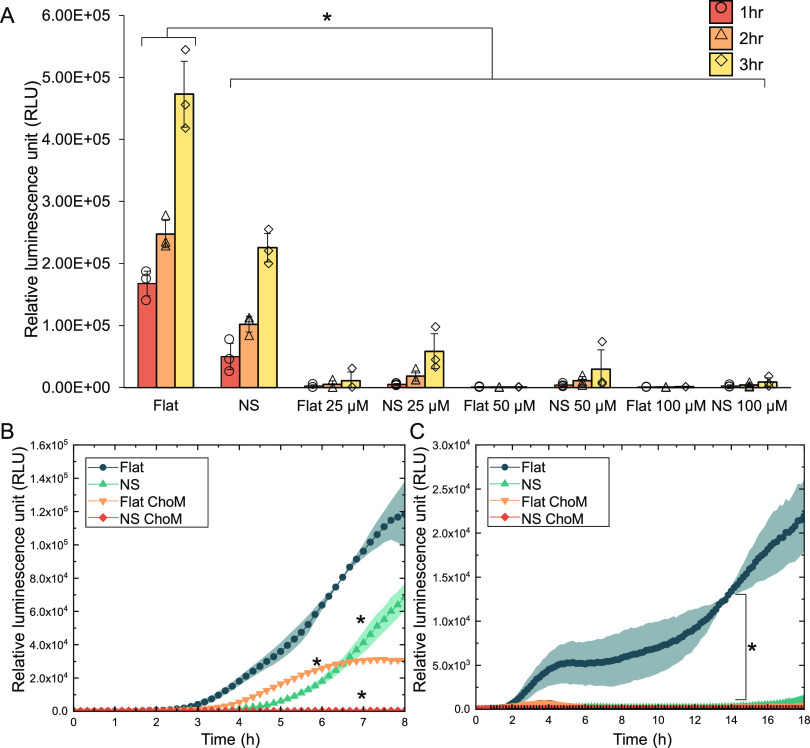
Viability of bacteria on different functionalized NS surfaces. The antibacterial activity of titanium surfaces functionalized with 0–100 μM ChoM was assessed against (A) *E. coli* over 3 h using BacTiter-Glo or (B,C) using RealTime-Glo against (B) *S. aureus* over 8 h or (C) *S. epidermidis* over 18 h. Data are presented as mean ± SD. *P < 0.05 compared to the control as determined by one-way ANOVA with Tukey HSD post hoc test; *n* = 3.

After the first hour of incubation with *E. coli*, there was a 70% reduction in viable bacteria on the NS surface when compared to the flat surfaces. Cell numbers increased on both nonfunctionalized surfaces up to 3 h, indicating some degree of bacterial growth, but the significant difference in viability was maintained, confirming the antibacterial properties of the NS surface alone. When functionalized with 25 μM ChoM, the flat surface showed significant antibacterial activity after 1 h of incubation compared to the control surface and bacterial growth continued at a limited rate after 3 h. When the flat surface was functionalized with peptide at the higher concentrations, total inhibition of *E. coli* growth was observed over the 3-h incubation period (Figure 3A).

There was a significant reduction in bacterial growth for the NS-25 μM surface relative to the control and NS surfaces after 1 h, but no significant difference was found when compared to the Flat-25 μM surface. The bacteria were still able to proliferate on the NS-25 μM surface over the 3-h period but at a significantly slower rate compared to the nonfunctionalized surface. After 3 h, there was a 75% reduction in cell viability for the functionalized NS surface compared to the nonfunctionalized surface. In contrast to the flat surface, when the NS surface was functionalized with higher peptide concentrations (i.e., 100 μM), *E. coli* still managed to grow after 3 h although at a much slower rate.

These studies were extended to assess the antibacterial performance of the NS surface functionalized with 100 μM ChoM against Gram-positive species, specifically *S. aureus* and *S. epidermidis*. These studies were run using the RealTime-Glo assay until growth of the population started to decline, which was after approximately 8 or 18 h, respectively (Figure 3B,C). On the nonfunctionalized flat titanium surface, the luminescence signal generated by *S. aureus* increased over 8 h, reaching a maximum of 1.2 × 10^5^ RLU. As before, the presence of nanospikes significantly impaired *S. aureus* growth relative to control, with a maximum RLU of 7 × 10^4^ reached after 8 h. Following functionalization, a slower level of growth was seen on the flat surface compared to the nonfunctionalized control, reaching a plateau between 6and 7 h at a RLU of 3.0 × 10^44^. By contrast, functionalization of the nanospikes with ChoM ablated *S. aureus* growth over the 8 h period. *S. epidermidis* displayed an even greater susceptibility to the nanostructures in the presence of ChoM. Upon incubation on the NS, Flat-ChoM, and NS-ChoM surfaces, no growth was detected over the 18-h period, whereas *S. epidermidis* growth on the flat, nonfunctionalized control reached an RLU of 2.3 × 10^4^ after 18 h.

### 3.3 Biocompatibility of Peptide-Functionalized Surfaces

Antibacterial strategies tend to also be detrimental to eukaryotic as well as prokaryotic cells.^18^ Therefore, an additional aspect of these studies was to determine any reduction in biocompatibility and osteogenic potential of the NS surface following functionalization with ChoM. This is an important consideration for the intended application as an implant material as host cell adhesion to the implant material is required for long-term infection control in winning the “race to the surface.”^19^

**Figure 4:**
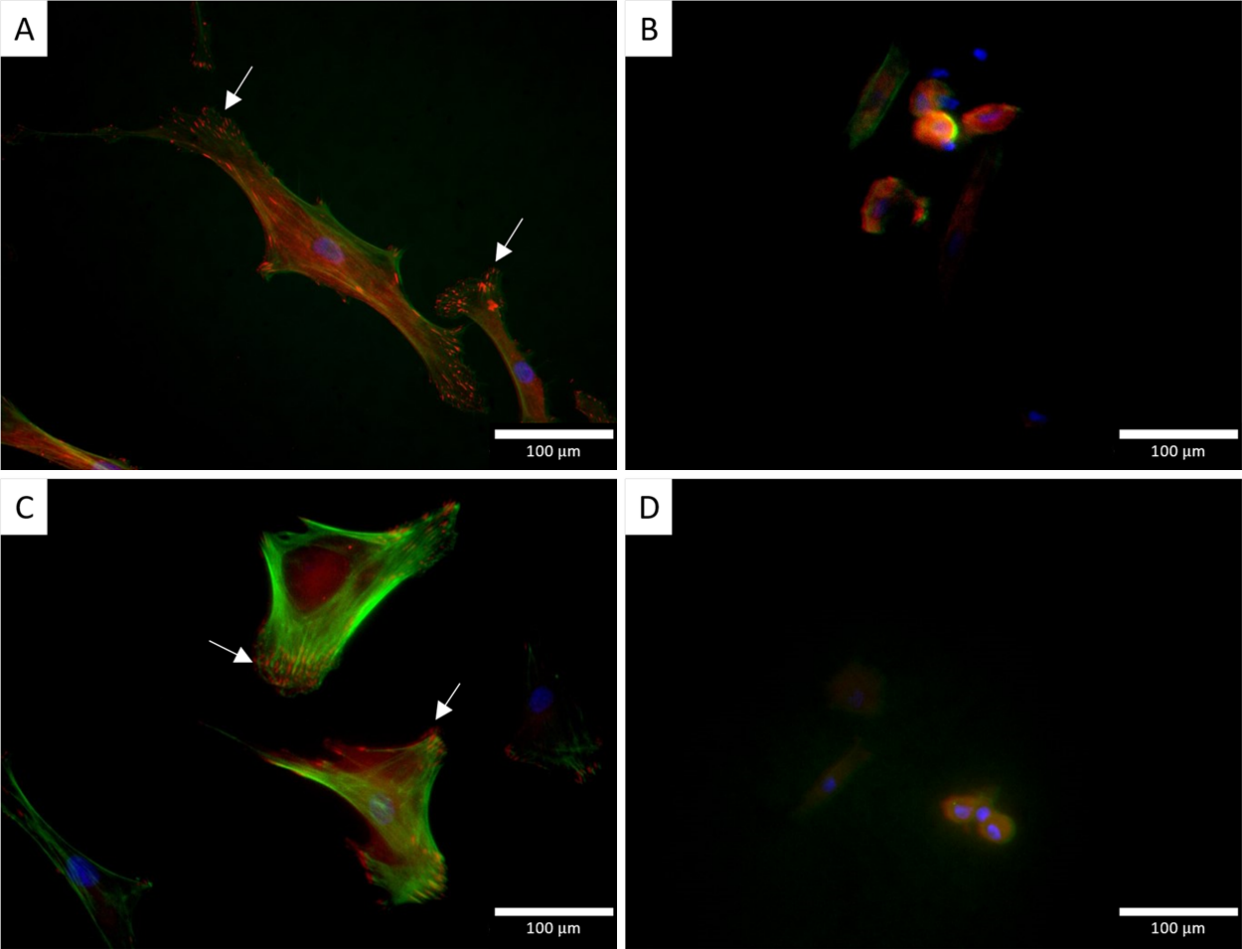
Immunofluorescent staining of hMSCs after 3 days on different surfaces. hMSCs were seeded onto (A) nonfunctionalized flat, (B) nonfunctionalized NS, (C) ChoM-coated flat, and (D) ChoM-coated NS surfaces at 3000 cells/cm^2^ and incubated for 3 days at 37 °C in 5% CO_2_. Cells were washed, fixed, and then stained for vinculin (green) and actin (red). Nuclei were stained with DAPI (blue). White arrows indicate actin focal points.

To determine if ChoM functionalization of the nanospikes affected human mesenchymal stem cell (hMSC) adherence to the titanium surfaces, immunofluorescent staining was used to visualize focal adhesion formation. Cell elongation and a well-organized cytoskeleton were visualized on the flat titanium disks in the presence or absence of ChoM (Figure 4A). Numerous focal adhesion points could be seen (in red and highlighted with white arrows), indicating attachment to the titanium surfaces. These adhesion points were observed along the leading edges of the cells, highlighting the movement of the cell in multiple trajectories across the surface. Lamellipodia and filopodia were also present as the cells spread on the surface. On the NS surface, smaller hMSCs were observed with less evidence of cell spreading or motility across the surface (Figure 4B). These images suggest that after 3 days, the hMSCs preferred to attach and spread on flat titanium rather than the nanospikes. The presence of the ChoM coating had no discernible effect on these interactions. This slower adhesion has been observed before on high aspect-ratio nanotopographies,^20^ but it is important that the hMSCs did adhere and the ChoM did not have further detrimental effects.

**Figure 5:**
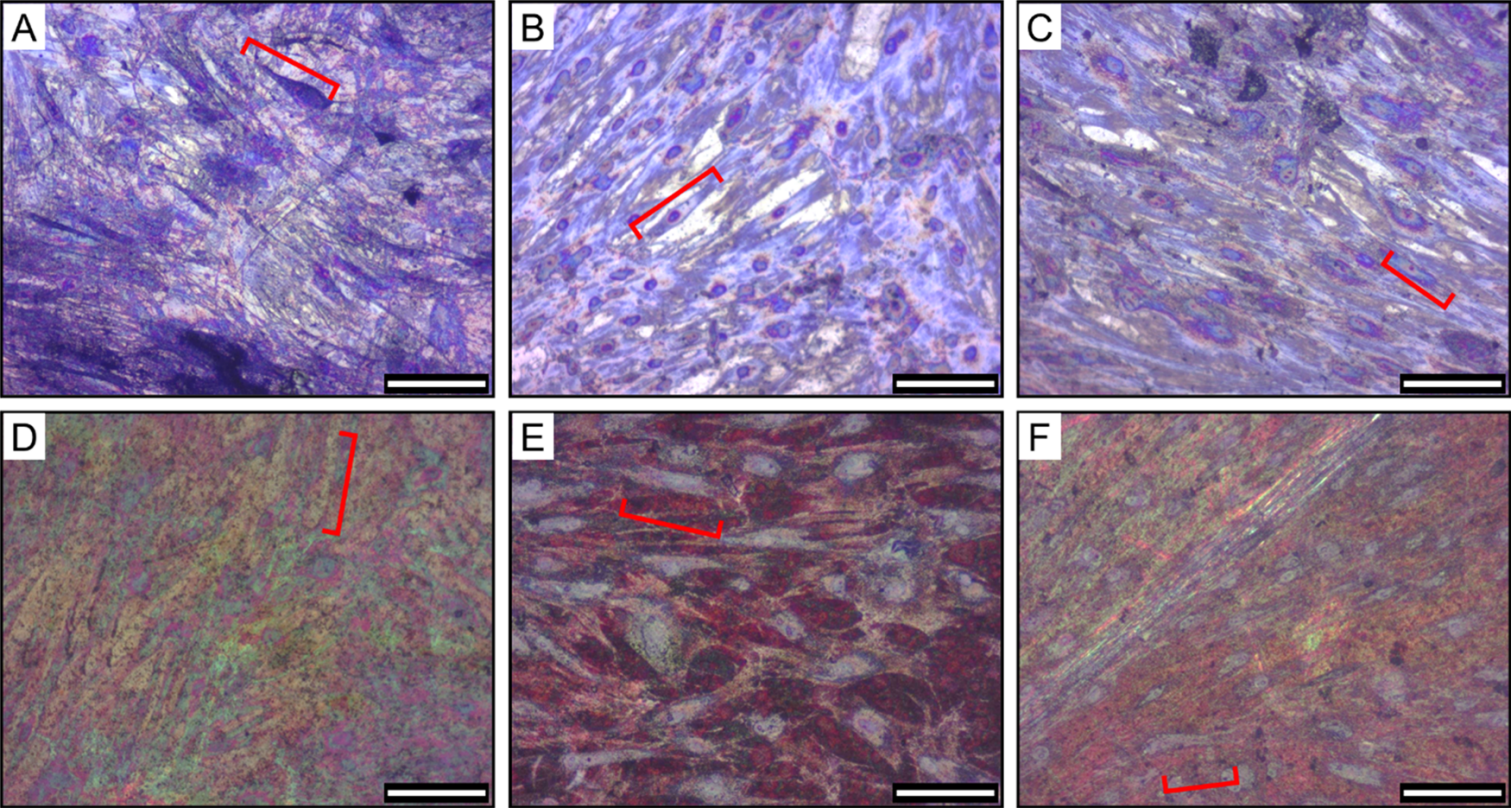
Brightfield imaging of Giemsa-stained hMSCs on different surfaces. hMSCs were seeded onto (A) nonfunctionalized flat (positive control), (B) nonfunctionalized flat, (C) ChoM-coated flat, (D) nonfunctionalized NS (positive control), (E) nonfunctionalized NS, and (F) ChoM-coated NS titanium disks. The disks were seeded with 3000 cells/cm^2^ and incubated for 28 days at 37 °C in 5% CO_2_. Positive control disks (A,D) were incubated in osteogenic medium. Cells were washed, fixed, and then stained with Giemsa stain. Red brackets highlight individual cells. Scale bar, 200 μm.

We also utilized Giemsa staining to visualize and assess the morphology and confluency of the hMSCs on the tested surfaces after the 28-day incubation period. Osteogenic medium was used as a positive control to provide an environment to support hMSC adhesion and proliferation on both flat titanium and NS surfaces (Figure 5A,D). After 28 days on the nonfunctionalized flat titanium (Figure 5B), the hMSCs were clearly visible with the nuclei stained dark blue/purple and the cytoplasm a light blue. The cells had formed a dense layer with a range of morphologies, and the majority of cells demonstrated spreading and motility across the surface with elongated and stretched membranes. The morphology was similar to hMSCs grown in osteogenic medium (Figure 5A). The presence of ChoM did not adversely affect growth (Figure 5C).

Visualization of cells on the nanospikes with the light microscope was challenging due to the underlying color of the disks (Figure 5D–F), which was not seen on the flat surface. There was also high intradisk and interdisk variability in color and patterning. Nonetheless, a dense coverage of hMSCs was seen across the nonfunctionalized NS surface with cell growth, stretching, and motility evident for the majority of cells (Figure 5E). There was no observable difference in morphology compared to the cells grown in osteogenic medium (Figure 5D), and cells were comparable when grown on NS disks with the ChoM peptide coating (Figure 5F). Together, these results suggest that the hMSCs could adhere, grow, and spread across the nanospikes, as well as on the flat surface, regardless of the presence of ChoM. In all instances, however, no significant osteogenic differentiation occurred during the 28-day time period.

**Figure 6:**
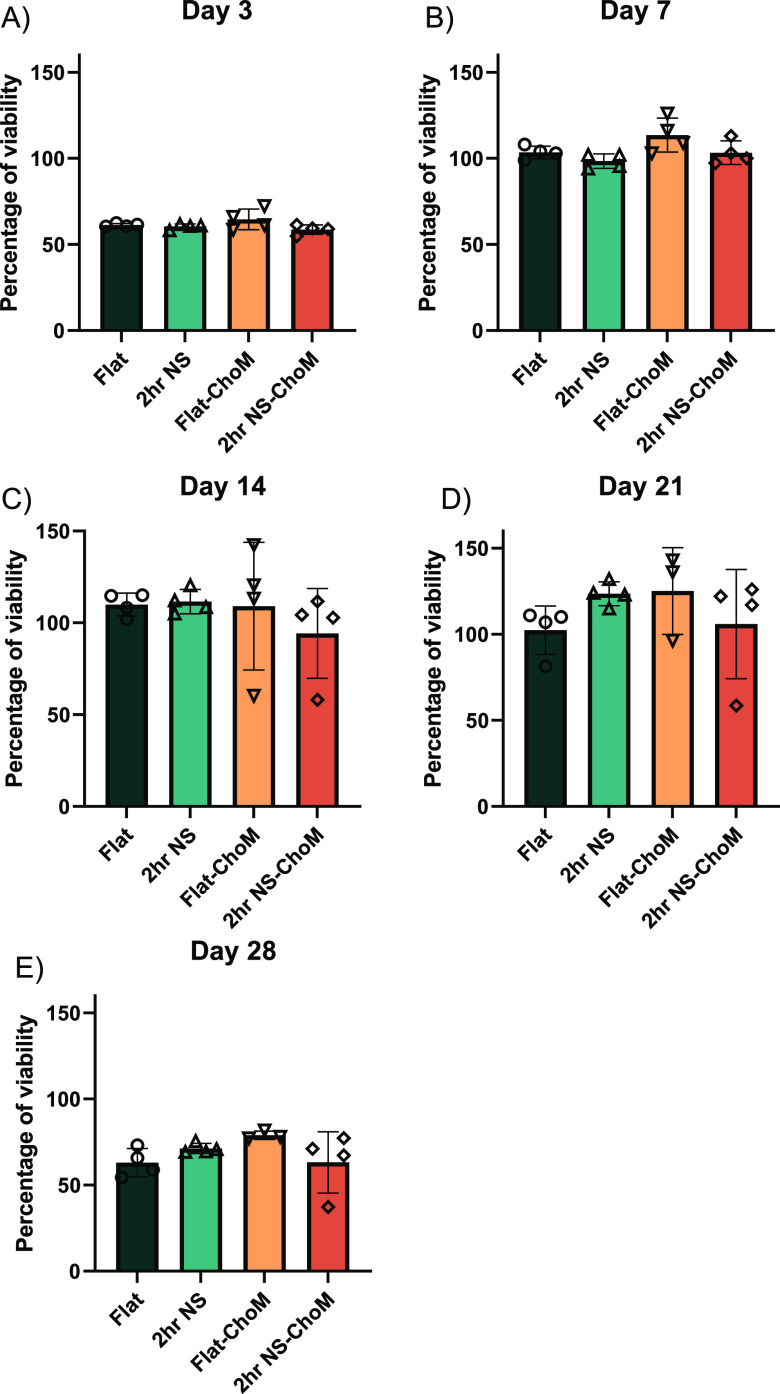
hMSC viability following incubation over 28 days on different surfaces. hMSCs were seeded onto flat and NS surfaces ± 100 μM ChoM and incubated at 37 °C in 5% CO_2_ for (A) 3 days, (B) 7 days, (C) 14 days, (D) 21 days, and (E) 28 days. AlamarBlue solution was then added to each well and incubated for 6 h at 37 °C in 5% CO_2_, and the absorbance measured at 570 and 600 nm. Data are presented as mean ± SD; *n* = 4 in quadruplicate.

To investigate the biocompatibility of the surfaces over the extended 28 day period, the alamarBlue assay was used. This assay quantitatively determines the viability of mammalian cells by measuring the innate reducing power of a cell. Living cells take up and reduce resazurin into resorufin, which fluoresces red. On day 3, hMSC viability was around 60% on both the flat and NS surfaces with or without ChoM functionalization (Figure 6A). A similar trend was seen at days 7, 14, and 21 with cells on all surfaces exhibiting viability values of ∼100% (Figure 6B–D). In 28 days, the longest timepoint was tested, and the viability dropped slightly for all the surfaces to approximately 70% (Figure 6E). Again, there was no statistically significant difference between the surfaces ± ChoM, indicating that the peptide did not cause any cytotoxicity. These results demonstrate that while initial hMSC attachment dynamics were slower on the NS surface compared to flat, both surfaces ultimately exhibited comparable biocompatibility, at least over a 28-day period, and were not adversely affected by the ChoM peptide coating.

## 4 Discussion

To the best of our knowledge, this is the first report on enhancing the antibacterial properties of a TiO_2_ nanotopography by biomimetic. Prior efforts to enhance the antibacterial performance of nanospikes have been through the use of incorporated metal ions like copper, zinc, silver, and magnesium.^21−25^ In this study, we chose to functionalize the titanium surfaces with AMPs, which unlike metal ions are biodegradable and nontoxic to human cells. Similar to antimicrobial metal ions, resistance toward AMPs by bacteria is limited.^26^

### 4.1 Release Kinetics of ChoM

Physical adsorption was chosen as the method of functionalization because it is a simple technique with no special surface treatment needed, thereby avoiding the use of linkers such as glutaraldehyde that are potentially cytotoxic.^27^ Physical adsorption methods allow the free release of AMPs into the local environment from the surface, enabling the peptides to exert effective, short-term antimicrobial activity. In these studies, ChoM was rapidly released within 10 min from the flat titanium surface and reached a higher concentration than achieved with the NS surface over 3 h. However, SEM images of the disks indicated that while only a residual peptide coating was left on the flat surface after 3 h, significant peptide remained on the NS surface, implying that ChoM release from the nanospikes could be maintained for a much longer period. This was supported by subsequent studies exploring the antibacterial properties of the surfaces, in which the functionalized NS surface was more effective against *S. aureus* than the equivalent flat surface over 8 h.

**Figure 7:**
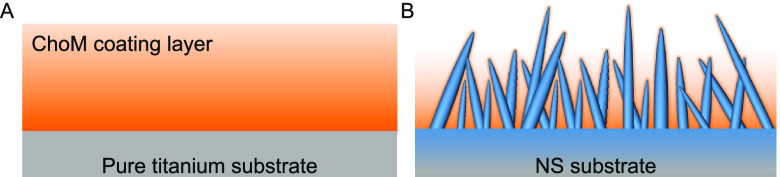
Schematic of the differences in ChoM coating found on (A) flat control and (B) NS surfaces.

Adsorption and desorption processes are dependent upon factors, such as surface chemistry, physicochemical properties of the solvent and the surface, surface area, and topography.^28,29^ Given the differences observed with the flat mirror polished titanium surface and NS surface, it is anticipated that ChoM release dynamics were influenced by one or more of these properties (Table S2). Wettability is the most significant difference between the two surfaces where the flat surface is slightly hydrophilic with a contact angle of 80°, while the NS surface has high wetting with a contact angle of less than 10°. The superhydrophilicity of the NS surface could be due to an increase in surface roughness and total surface area, which together lead to a higher overall surface energy.^30,31^ Roughness promotes the spread of the liquid, while the large surface area of the nanotopography further enhances the surface wetting.^32^ The volume of ChoM that was used to functionalize the surfaces was limited by the maximum volume that could be pipetted onto the NS surface. Thus, the differences in surface wettability meant that the application of the same volume of ChoM led to differences in the coverage of the peptide coating on the two surfaces. A small, peptide-dense area formed on the flat surface, while the peptide solution on the superhydrophilic NS surface covered the entire surface (Figure 7). This variation in ChoM distribution explains why SEM showed the coating on the NS surface to be much thinner (<500 nm) than that on the flat surface and why XPS spectra confirmed significantly more amine groups on the flat surface compared to the NS surface.

SEM and XPS data suggest that the NS architecture directly influenced the adsorption and release kinetics of ChoM. The presence of nanospikes increases the total surface area of the titanium disk and provides a reservoir (Table S2). Consequently, most of the peptides were physically absorbed to the nanospikes and not readily released upon activation by growth medium or water (Figure 2E). This contrasts with the flat surface where most of the peptides were activated and released unimpeded into the suspension within a short period of time.

### 4.2 Antimicrobial Activity of Functionalized ChoM

It was initially anticipated that functionalization with ChoM would enhance the antibacterial properties of the NS surface. However, against *E. coli*, the efficacy of ChoM, even at 25 μM, was so high that the presence of this peptide alone was sufficient to kill the bacterial population over a 3-h period, independent of the nanospikes. However, after 3 h, it was only on the functionalized NS surface that slight bacterial growth was detected. Pyne et al. proposed that a monolayer pore-forming peptide could bind to a bacterial cell membrane in either the S-state (inactive state) or M-state (pore formation state) and that the antibacterial activity is folding-dependent. The peptides could also exfoliate the bacterial cell membrane without inserting into it, thus causing a more rapid and extensive membrane rupture.^14^ Thus, it is possible that the slight variation seen in antibacterial performance of the functionalized flat versus NS surfaces reflects the fact that ChoM residues that remained adsorbed to the nanospikes were not in a fully active conformation compared to the released form. Nonetheless, with longer incubation periods when testing the surfaces against Gram-positive species, there was evidence of the nanospikes and ChoM working together for enhanced bacterial killing. Growth of *S. epidermidis* was ablated over a 18-h period by either nanospikes or ChoM alone. However, for *S. aureus*, the presence of nanospikes or ChoM could only suppress growth for up to 3.5–4 h. By contrast, the combination of nanospikes and ChoM ablated *S. aureus* growth for the entire 8-h period. To more readily assess the contribution of nanospikes and ChoM on bacterial viability when alone or in combination, these data were also expressed as percentage change in bacterial viability for the NS, Flat + ChoM (100 μM), or NS + ChoM (100 μM) surfaces at the end of each assay compared to the flat control surface (Table S3). As predicted, the high susceptibility of *E. coli* to ChoM effectively masked any potential enhanced effect with nanospikes, with a >98% reduction in *E. coli* viability seen on either the Flat + ChoM or NS + ChoM surface. A similar situation was observed for *S. epidermidis* with either nanospikes or ChoM alone capable of reducing bacterial viability by >97%. By contrast, for *S. aureus*, nanospikes alone could reduce bacterial viability by 39% while ChoM alone resulted in a 73% reduction in viability. Combined, the reduction in *S. aureus* viability exceeded 99%, clearly demonstrating the additive effects of nanospikes with ChoM. This additive effect against *S. aureus* is likely important as it is Gram-positive pathogens such as these that are typically introduced by surgery, i.e., that are in the “race to the surface.”^19^

The combined action of nanospikes and ChoM is similar to Bright et al., who reported that exposure of bacterial cells incubated with nanospikes prior to vancomycin treatment mediated synergistic effects.^33^ Specifically, the interaction between bacteria and nanospikes caused intracellular reactive oxygen species (ROS) generation and bacterial upregulation of catalase in response. In the presence of vancomycin, however, upregulation of catalase was impaired, leading to elevated oxidative damage and resultant bacterial cell lysis.

**Figure 8:**
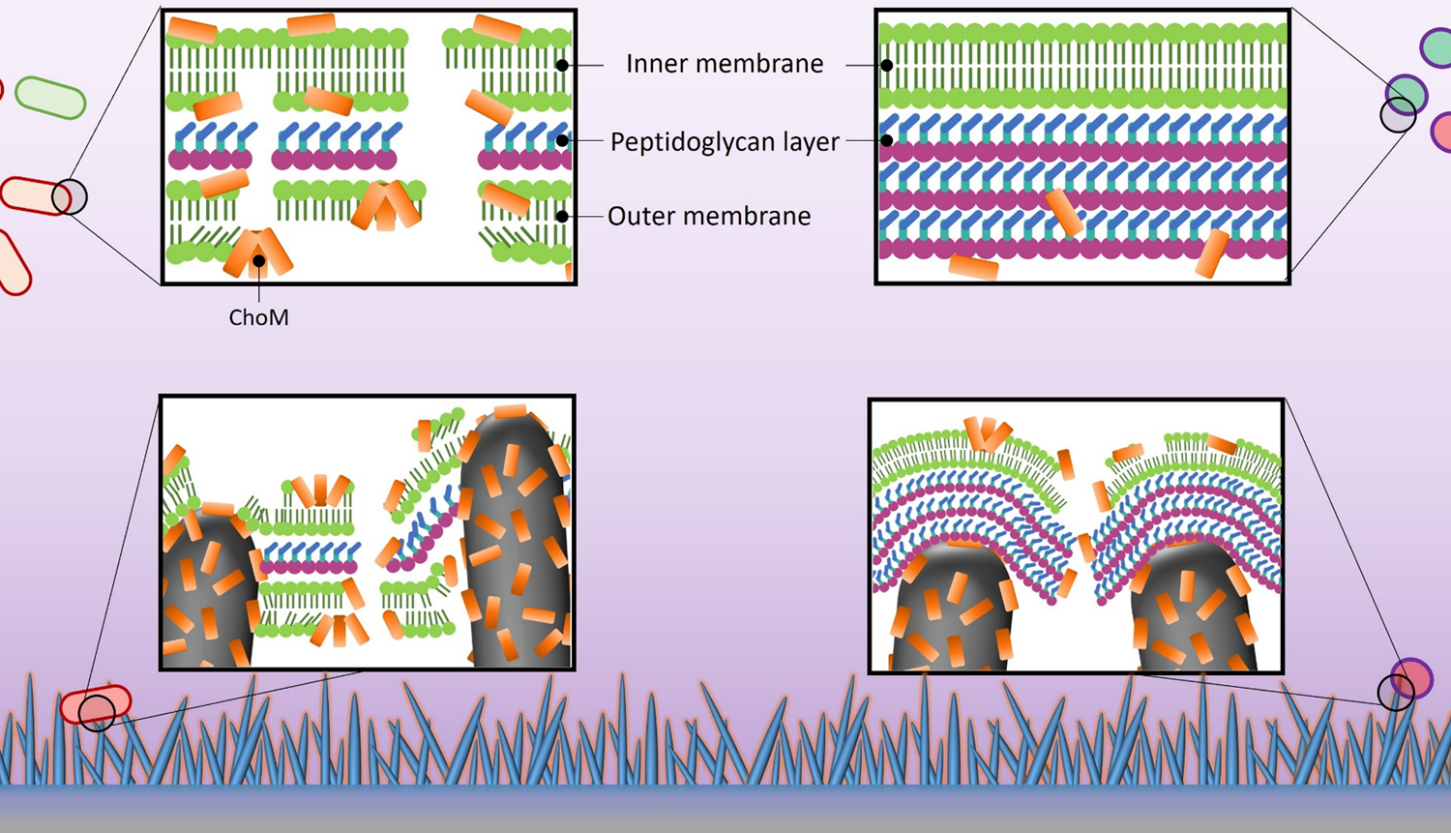
Schematic of proposed mechanisms of additive antibacterial effects between nanospikes and AMPs against Gram-negative and Gram-positive bacteria. Top insets show ChoM (orange cylinder) exfoliating the cell envelope of Gram-negative (left) and Gram-positive (right) bacteria. ChoM can rapidly start to compromise the outer and then inner cell membranes of Gram-negative bacteria, inducing cell lysis. For those bacteria that survive this initial challenge, the compromised cell membranes make them more susceptible to the sharp nanospikes, which can result in further membrane thinning and potential rupturing and penetration (bottom left inset). The presence of the thick peptidoglycan layer in Gram-positive bacteria prevents easy access of ChoM to the underlying cell membrane. However, bacterial contact with the nanospikes can stretch and compromise the peptidoglycan layer, thereby generating an opening for ChoM to access and disrupt the cytoplasmic membrane (bottom right inset). *The insets are not drawn to scale.

In proposing a potential mechanism for the additive antibacterial effects of nanospikes and ChoM, it is likely that the prolonged release of ChoM from the nanospikes relative to the flat surface is a factor, but it is also important to consider the nanospike–bacterium interface. Evidence from FIB-SEM has shown that adherent bacteria on nanospikes will undergo membrane stretching,^34^ which in turn induces the formation of ROS.^17,33,34^ ChoM mediates its antimicrobial effects by intercalating with phospholipid head groups within the lipid bilayer of the bacterial cell membrane. This leads to thinning and exfoliation of the lipid bilayer and ultimately cell lysis.^16^ For Gram-negative bacteria, it is expected that within the first 10 min of incubation, ChoM that is released from the coating starts to irreversibly perturb the outer lipid bilayer. This thinning and pore formation within the outer membrane then makes any surviving bacteria more susceptible to the effects of the nanospikes upon bacterial adhesion to the surface. Since the outermost layer of the Gram-positive bacterial cell envelope comprises a thick layer of peptidoglycan, ChoM cannot readily access the underlying phospholipid bilayer. However, on the functionalized NS surface, we propose that stretching of the adherent Gram-positive cell envelope by the nanospikes serves to expose the cytoplasmic membrane to ChoM and so enhance its bactericidal efficacy (Figure 8). This is a similar principle to a study in which cells became more susceptible to nanopillars after their membrane integrity had been compromised with microwave radiation.^35^

It is important to note that while debris resulting from damage to bacterial cells, such as proteins, may adsorb onto the NS surface, these constituents are unlikely to exhibit long-term stability and can be expected to degrade over time.^36^ Moreover, for the in vivo application of this functionalized NS surface, both hMSCs and microbial cells will be present simultaneously. The ultimate goal for the enhanced antimicrobial performance of the functionalized nanospikes is therefore to restrict microbial attachment and growth to a sufficient level that allows hMSCs to win the “race to the surface.” Once attachment and spreading of hMSCs is underway, microbial infection will be inhibited.

### 4.3 Biocompatibility of ChoM-Functionalized Nanospikes

In this study, hMSCs initially favored attachment to the flat titanium surface compared to the NS surface. This could be related to the diameter of the nanospikes as reported previously.^20,37,38^ In a study comparing nanospikes of different diameters, Goreham et al. reported that nanospikes with a 16 nm diameter encouraged adhesion of MG63 and 3T3 cells compared to nanospikes with 38 or 68 nm diameters.^39^ Sjöström et al. also reported that more adhesion and bone matrix formed on nanospikes with a smaller diameter (28 nm) compared to larger ones (41 and 56 nm).^40^ Nonetheless, over a longer time period, the NS surface was found to be of comparable biocompatibility as the flat surface. Greater levels of cellular adhesion on the nanospikes were observed over time, likely due to the deposition of serous and extracellular matrix (ECM) proteins onto the nanospikes, making the surface more favorable for hMSC attachment. Critically, as seen from our Giemsa and immunofluorescent staining results, functionalization with ChoM did not compromise the biocompatibility of the surfaces. This aligns with Pfeil et al., in which ChoM at a concentration of 250 μM was found to have no adverse effects on blood cells.^16^ Similarly, PEEK substrates that were functionalized with AMP and osteogenic growth peptide (OGP) showed resistance toward bacterial infection, stabilized bone homeostasis, and facilitated osteogenesis in vivo after 14 days.^41^ More recently, Gao et al. reported that TiO_2_ nanospikes functionalized with cationic polymers can kill both Gram-negative and Gram-positive bacteria and inhibit biofilm formation for up to 14 days. They also reported that the residual hydroxyl group on the titanium substrate promoted deposition of hydroxyapatite in Kokubo’s simulated body fluid, which was important for orthopedic and dental applications.^42^

## 5 Conclusions

In summary, this study has shown that a nature-inspired NS surface with a high density of high aspect ratio nanospikes can exhibit promising antibacterial effects against *E. coli*, *S. aureus*, and *S. epidermidis*. Moreover, the antimicrobial activity of the NS surface can be enhanced by functionalization with a bioinspired AMP, ChoM. For Gram-negative bacteria, we propose that these enhanced effects result from membrane stretching and deformation of the bacterial cell envelope by nanospikes following initial disruption of the outer cell membrane by ChoM. For Gram-positive bacteria, such as *S. aureus*, the thick peptidoglycan layer of the cell envelope renders them less susceptible to the immediate effects of ChoM. In this instance, the additive effects come from interactions between adherent bacteria and nanospikes that cause the cell wall to experience deformation, which in turn generates openings for the ChoM to access the underlying cytoplasmic membrane. Crucially, when considering their potential as novel implant materials, both the functionalized and nonfunctionalized surfaces were found to be highly biocompatible. Taken together, this research highlights the potential to generate biocompatible titanium surfaces with enhanced antibacterial activity comprising both physical and chemical mechanisms of action. Such an approach could be exploited to develop next-generation implants to combat bacterial infections, and thus maximize the longevity of medical implants and improve the wellbeing of millions of patients worldwide.

## Supporting Information Available

XPS spectra of pure titanium and NS surfaces (Figure S1); bacterial strain information (Table S1); surface topography and wettability data for flat control, NS, and dragon fly wing surfaces (Table S2); and % change in cell viability for bacteria incubated on NS, Flat + ChoM, and NS + ChoM surfaces relative to flat control (Table S3) (PDF)
