# Supplementary material for: Enhanced and Stem-Cell-Compatible Effects of Nature-Inspired Antimicrobial Nanotopography and Antimicrobial Peptides to Combat Implant-Associated Infection
Source: ACS Appl Nano Mater. 2023 Feb 15;6(4):2549–59. doi: 10.1021/acsanm.2c04913 (PMC9972347; doi:10.1021/acsanm.2c04913)
Supplement: Supplementary file 1 — an2c04913_si_001.pdf [file an2c04913_si_001.pdf]

Supporting Information

Enhanced and Stem-Cell Compatible Effects of Nature-Inspired Antimicrobial Nanotopography and Antimicrobial Peptides to Combat Implant-Associated Infection

Mohd Irill Ishak[1], *Marcus Eales*[1,2], *Laila Damiati*[4], *Xiayi Liu*[1], *Joshua Jenkins*[1], *Matthew J. Dalby*[3], *Angela H. Nobbs*[1], *Maxim G. Ryadnov*[2], *Bo Su*[1*]

[1]Bristol Dental School, University of Bristol, Bristol, BS1 2LY, UK

[2]National Physical Laboratory, Teddington, London, TW11 0LW, UK

[3]Centre for the Cellular Microenvironment, University of Glasgow, Glasgow, G11 6EW, Scotland, UK

[4]Department of Biology, College of Science, University of Jeddah, Jeddah 23218, Saudi Arabia

[*]Email: b.su@bristol.ac.uk

Supplementary Experimental Procedures

Atomic Force Microscopy (AFM)

Nanospikes were imaged and the average height, surface area and RMS (average-root mean square) roughness were quantitatively measured using AFM. Nanospikes were imaged using an AFM (Digital Instruments INC Nanoscope IIIa Atomic Force Microscope) and contact probe tips (MikroMash). An area of 25 μm^2 with a scan rate of 0.5 Hz and 512 lines was measured. The average roughness (R_a), maximum peak-to-valley heights (R_{max}), and surface area (Table S2) were quantified using Gwyddion v2.56.

Supplementary Figure

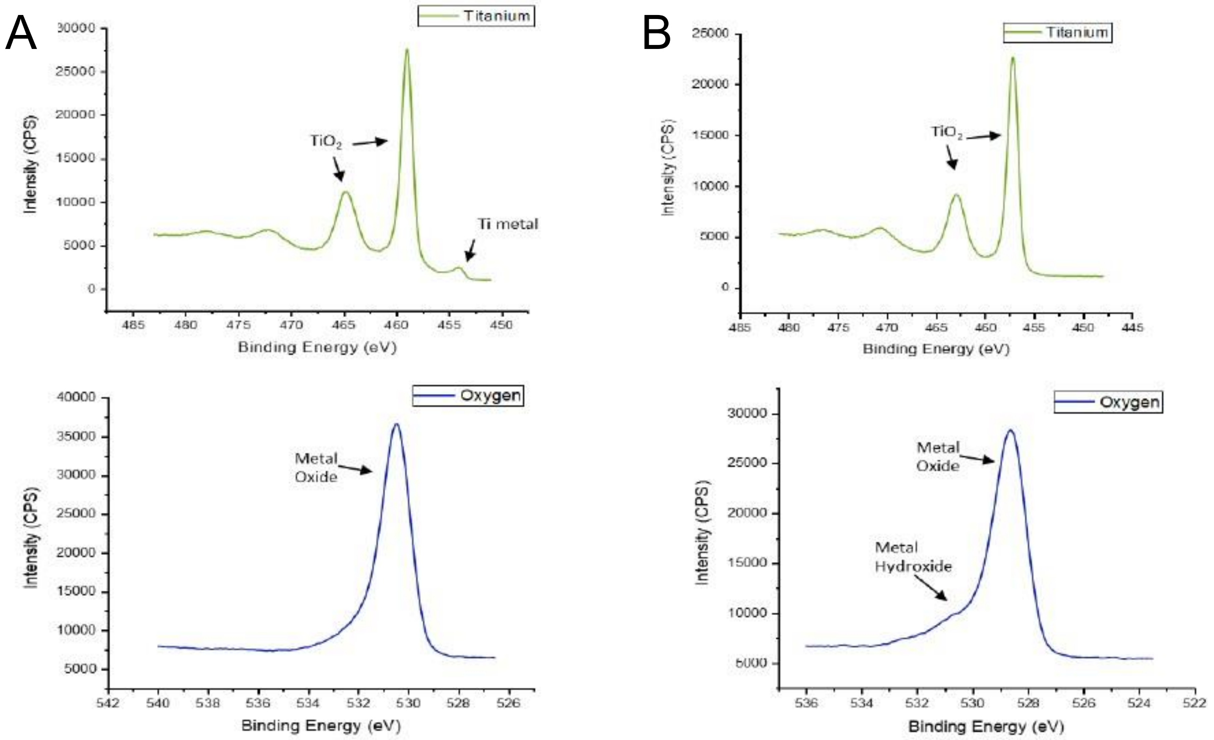

Figure S1. XPS spectra of pure titanium (A) and NS (B) surfaces.

Supplementary Tables

Table S1. Bacterial strains used and relevant characteristics.

Strain ID	Bacterial Strain	Morphology	Gram Identity	Motility	Reference / Source
UB 776	*Escherichia coli* DH5α	Bacillus	Gram negative	Motile	ThermoFisher Scientific
UB 1812	*Staphylococcus epidermidis* RP62A	Coccus	Gram positive	Non-motile	1
UB 1621	*Staphylococcus aureus* Newman	Coccus	Gram positive	Non-motile	2

Table S2. Physical parameters of flat control and nanospike (NS) surfaces measured using SEM, AFM, and drop shape analyser.

	Diameter (nm)	Height (nm)	Density (NS/nm)	Wettability (º)	R_q	R_{max}	Surface Area (μm^2)
Control	N/A	N/A	N/A	82±2.1	3.1±0.2	62.6±2.1	25±4.5
NS	40±10.2	440±90	36.6±4.4	<10	112.3±10.5	686±20.8	61.8±8.3
Dragon fly wing*	47.4	433.4	74.2	157	170.7	N/A	N/A

R_a=average roughness, R_{max} = maximum peak-to-valley heights; *from [3]

Table S3. Percentage change in cell viability for bacteria incubated on NS surfaces ± ChoM (100 μM) or flat surface + ChoM (100 μM) relative to bacteria incubated on control flat surface.

Strain	NS only	Flat + ChoM	NS + ChoM
E. coli	-52.3±0.8%	-99.8±0.0%	-98.0±2.0%
S. aureus	-38.8±23.4%	-73.2±7.3%	-99.7±0.0%
S. epidermidis	-97.4±1.0%	-98.4±0.4%	-99.8±0.2%

The percentage change was calculated using Equation S1, where V_2 is the test value, and V_1 is the control value.

$$\%\ change = \frac{(V_2 - V_1)}{V_1} \times 100 \qquad \text{Eq. S1}$$

REFERENCES

(1) Christensen, G. D.; Simpson, W. A.; Younger, J. J.; Baddour, L. M.; Barrett, F. F.; Melton, D. M.; Beachey, E. H. Adherence of Coagulase-Negative Staphylococci to Plastic Tissue Culture Plates: A Quantitative Model for the Adherence of Staphylococci to Medical Devices. *J. Clin. Microbiol.* **1985**, *22* (6), 996–1006.

(2) Duthie, E. S.; Lorenz, L. L. *Staphylococcal Coagulase*: Mode of Action and Antigenicity. *Microbiology* **1952**, *6* (1–2), 95–107.

(3) Truong, V. K.; Geeganagamage, N. M.; Baulin, V. A.; Vongsvivut, J.; Tobin, M. J.; Luque, P.; Crawford, R. J.; Ivanova, E. P. The Susceptibility of *Staphylococcus aureus* CIP 65.8 and *Pseudomonas aeruginosa* ATCC 9721 Cells to the Bactericidal Action of Nanostructured *Calopteryx Haemorrhoidalis* Damselfly Wing Surfaces. *Appl. Microbiol. Biotechnol.* **2017**, *101* (11), 4683–4690.
